# Adding home-use BIA scales, online food diaries, and tape-measures to large-scale questionnaire studies: Insights from the population-based PROFILES registry research

**DOI:** 10.1007/s00520-026-10536-x

**Published:** 2026-03-09

**Authors:** Floortje Mols, Nicole P. M. Ezendam, Sandra Beijer

**Affiliations:** 1 CoRPS-Center of Research On Psychological Disorders and Somatic Diseases, Department of Medical and Clinical Psychology, Tilburg University, Tilburg, The Netherlands; 2 Department of Research & Development, Comprehensive Cancer Organisation (IKNL), Utrecht, The Netherlands; 3 Department of Dietetics, Maastricht University Medical Center+, Maastricht, the Netherlands

**Keywords:** Cancer, Home-use BIA scale, Food diary, Ambulant, Self-measures, PROFILES

## Abstract

With the rising prevalence of cancer, longitudinal research on survivorship increasingly emphasizes patient-reported outcomes (PROs). Traditionally, these outcomes have relied on self-report questionnaires and clinical data, which provide valuable insights but may not fully capture underlying biological and physiological processes. To bridge this gap, the PROFILES registry was recently expanded to incorporate objective, home-based measures that participants can perform independently, such as food diaries, body composition scales, and activity trackers. This paper summarizes lessons learned from implementing home-use bioelectrical impedance analysis (BIA) scales, online food diaries, and tape measures to assess body composition and nutritional intake in large-scale ambulatory studies. Such studies, conducted in participants’ everyday living environments rather than clinical settings, provide ecologically valid insights but also raise methodological and practical challenges. When selecting self-monitoring tools for research, reliability and validity remain essential, yet other considerations are equally important. Widely used tools facilitate comparability and generalizability across studies, while feasibility, practicality, sensitivity to change, and standardization determine their practical value. Clear instructions, efficient logistics, and user-friendliness support sustained participant engagement. Furthermore, legal and ethical requirements must be carefully addressed to ensure data privacy and compliance with regulatory standards. Integrating objective measures with PROs enhances accuracy, allows triangulation of self-reported and observed outcomes, and improves prediction of long-term survivorship trajectories. Since 2009, PROFILES data have been shared globally for non-commercial research, and upcoming expansions to include objective measures will further strengthen its impact. These experiences offer valuable guidance for future survivorship research and beyond.

## Introduction

Together with the rising prevalence of cancer, the number of large longitudinal population-based studies that follow cancer survivors from diagnosis into survivorship has increased [1]. Those studies, which are often solely based on questionnaire data complemented by clinical data, increase the knowledge on the course of physical and psychosocial outcomes during and after cancer and its treatment. However, knowledge on the possible underlying biological and physiological mechanisms of those outcomes is largely lacking. Accurately predicting the risk for poor physical and psychosocial outcomes among survivors is therefore currently challenging.

A first step, to not only identify cancer survivors at risk for poor physical and psychosocial outcomes, but to also understand the driving mechanisms behind this, is to complement studies that assess patient-reported outcomes (PROs) with other, more objective, measures. Since most PRO studies are based on self-report questionnaires, any additional measures should be feasible for ambulatory assessment. The most obvious and useful variables to assess in combination with PROs are potentially modifiable lifestyle factors, which offer valuable insight into behaviors that may influence recovery and long-term outcomes. Importantly, these factors are of interest not only from a scientific perspective, but also because they reflect areas where survivors themselves may take action to improve their health. Considering increasing pressure on healthcare systems, it is critical to identify strategies that help reduce the risk of cancer recurrence and long-term complications. Supporting survivors in adopting and maintaining a healthy lifestyle is a promising approach to achieving this goal.

In 2008, the dynamic population-based PROFILES (Patient Reported Outcomes Following Initial treatment and Long-term Evaluation of Survivorship) registry was set up. PROFILES is an infrastructure for interdisciplinary and longitudinal data collection of the physical and psychosocial impact of cancer and its treatment [2]. PROFILES collects PRO data from online and paper-and-pencil questionnaires and is linked to the Netherlands Cancer Registry (NCR) that collects clinical data of all Dutch cancer patients. It aims to describe and understand the possible impact of cancer and its treatment, beyond ‘normal’ aging and the presence of comorbidities. Within PROFILES, we use the American Cancer Society’s definition of cancer survivorship, which defines cancer survivors as those living with, through and beyond cancer [3].

PROFILES resulted in over 250 scientific papers on PROs among cancer survivors [1]. From 2016 onwards, the PROFILES registry was expanded with innovative data collection methods to boost mechanistic research to understand the driving mechanisms behind declining outcomes after cancer [4]. Since then, information from questionnaires and the NCR was enriched with novel, ambulatory and objective measures (i.e., online food diaries, home-use bioimpedance analysis (BIA) scales, body tape measures, activity trackers, hair samples, and blood draw) to create a multidomain data source for mechanistic cancer survivorship research [4]. This set-up was used in several PROFILES studies [5–7]. Insights from these studies will enhance patient education, screening for adverse physical and psychosocial outcomes, and the development of tailored treatments and supportive care to improve quality of life (QOL).

Incorporating objective and ambulant measures in several PROFILES-studies was challenging, and literature on how to incorporate these kinds of measures was largely lacking. Therefore, the aim of this paper is to reflect on the challenges we faced when including objective and ambulant measures to PROs studies, and to summarize our lessons learned. More specifically, in this paper, we will focus on home-use BIA scales to measure body weight and body composition, online food diaries to measure nutritional intake and tape measures to measure waist, hip, and calf circumference.

## What to consider when choosing the most suitable instrument for a study

*Reliability* and *validity* are important considerations when selecting objective and ambulant self-monitoring tools for a study. However, other issues should be considered as well. First, choosing *widely used tools* can be important since this allows researchers to compare findings across studies, contributing to a more cohesive body of evidence, which can improve generalizability and impact of the research findings. Additionally, it can facilitate peer acceptance of the research, as familiar tools are easier for others to evaluate and build upon in future studies. Second, *feasibility and practicality* are important, both for researchers and participants. Instruments should be easy to administer, require minimal time or training, and impose limited burden, making them manageable within the scope and resources of the study. In large cohorts such as the PROFILES registry, it is crucial to select tools that can be used independently at home. At the same time, population-specific appropriateness must be ensured. Instruments should fit the characteristics of the target population, considering factors such as age, cultural background, health status, and possible limitations. Tools that are intrusive, uncomfortable, or poorly aligned with participants’ needs may reduce engagement and adherence, ultimately affecting data quality [8]. Third, *sensitivity to change* is important in longitudinal studies or intervention research where changes over time are relevant. Fourth, *standardization and calibration* should be checked. Consistent application and regular calibration of the tools are essential for producing accurate, reproducible results. Fifth, *technological requirements and compatibility* are important. The necessary technology should be accessible, compatible with study requirements, and protocols should be in place for maintaining and troubleshooting it. Sixth, when setting up large studies, it is important to decide beforehand whether to buy one *big batch* of instruments of a similar version with the risk of not using them all when inclusion proves to be difficult. Or to buy fewer instruments at the start of your study with the risk of not being able to buy the exact instrument if you need more of them. Finally, *costs* and *legal matters* are important, which are described below. Balancing these considerations ensures the selected tools are robust, practical, and aligned with study goals. Below, we will describe the choices we made for our PROFILES studies regarding home-use BIA scales, food diaries, and body tape measures. Our lessons learned are summarized in Table 1.

**Table 1:** Lessons learned from adding home-use BIA scales, online food diaries, and tape measures to the PROFILES registry

Domain	Lesson	Practical tip
***Instrument selection***	Reliability and validity are essential, but not sufficient	Also check feasibility, sensitivity to change, standardization, and population-appropriateness
	Widely used tools enhance comparability, generalizability, and peer acceptance	Select instruments already validated and commonly used in similar studies
	Compatibility and technological requirements can hinder or facilitate data collection	Ensure tools work with participants’ devices and study infrastructure and that patients are able to understand and use them
	Purchasing strategy is important in large cohorts	Decide upfront: bulk purchase (risk of unused stock) vs. smaller batches (risk of different versions)
	Costs, legal, and ethical requirements must be anticipated	Consult legal experts and ethics committees before starting
***Body composition***	BMI alone is insufficient to assess body composition	Include waist, hip, and calf circumference for better insight
	Self-measured tape measures are valid and reliable	Provide clear written/visual instructions for consistent use
	Home-use BIA scales show good test–retest reliability but moderate validity	Use repeated measurements (e.g., daily over 2 to 4 weeks) and average results
***Food intake***	Online diaries reduce recall bias and participant burden, and ease researchers’ work by providing automatically calculated scores	Use validated web-based tools preferably linked to national nutrient databases
	Account creation and email confirmation were barriers	Provide step-by-step support; offer assistance during hospital visits when possible
	Motivation drops if diaries are placed at the end of long questionnaires	Send food diaries separately or integrate earlier in the study process
	Direct participant support improves adherence	Call participants before and after the diary completion; provide videos and helpdesk
***Legal and ethical***	Data storage by external providers requires agreements	Arrange processor agreements
	Ethical boards evaluate participant burden and instrument safety	Provide detailed study protocols including safety and feasibility
	Legal advice is necessary for compliance in each context	Involve legal experts early in study design
***Logistics***	Sending home-use BIA scales worked well but some were lost or not returned	Use pre-stamped return boxes and reminder systems
	Offering devices to participants may increase motivation	Consider allowing participants to keep devices (budget permitting)
	Distributing tape measures with study information was efficient	Purchase slightly more than target sample size
	External tool updates caused login issues	Anticipate technical updates and plan for troubleshooting support
***Data management***	Data from different sources require careful integration	Use secure APIs and standardized merging procedures; perform outlier checks
***Scientific value***	Combining PROs with objective measures enriches understanding	Use both subjective and objective data to improve predictions and reveal discrepancies
	Normative data is crucial for interpretation	Contextualize findings using national or international reference datasets
***Participant engagement***	Personal support strongly improves inclusion	Help participants create accounts in hospital settings; maintain contact via phone before and after participation

### Body composition

Anthropometric measurements are used in cancer survivorship research to assess the association between basic body metrics (e.g., height, weight, waist, and hip circumference) and clinical outcomes [9]. While body mass index (BMI) is often used as a proxy for overall body composition, it has important limitations. BMI does not differentiate between fat mass and lean mass and therefore does not provide sufficient insight into an individual’s body composition. This is particularly relevant in cancer survivors, as body composition may be severely impacted by cancer treatment [10, 11], which can lead to significant variations in fat and muscle distribution among survivors. Individuals with similar BMI values may vary greatly in fat distribution, muscle mass, and the presence of sarcopenia—an important prognostic factor associated with decreased physical function, poorer treatment tolerance, and reduced survival [12]. Our standard PRO questionnaires always contain questions on usual and current body weight and height so that researchers can assess BMI and weight changes. BMI correlates the risk of health problems with weight at population level. However, waist circumference or waist-to-hip ratio is a better estimate of visceral fat and therefore more appropriate to investigate relationships specifically between fat mass and outcome measures [13]. Furthermore, in February 2019, the international GLIM consortium (Global Leadership Initiative on Malnutrition) published a set of consensus criteria for diagnosing malnutrition in adults [14]. This set includes, besides disease burden, low BMI and weight loss, also low nutritional intake and reduced muscle mass. To determine the amount of muscle mass, the GLIM consortium recommends measuring calf circumference, upper arm circumference or, if possible, to perform bioelectrical impedance analysis, if advanced methods for determining muscle mass (such as DEXA or CT scan) are not possible (as in the PROFILES registry). Therefore, we added instructions and a tape measure to our PRO questionnaires so that patients can assess their waist, hip, and calf circumference themselves at home. Using tape measures by participants themselves is considered to be valid and reliable and can easily be done at home [15].

In addition, body composition is assessed using a home-use BIA scale with eight tactile electrodes (four foot and four hand contacts; InBody Dial H20N). We bought 20 scales of the same type at once. BIA is a double-indirect method used to estimate body composition. The Inbody scale measures the impedance (composed of resistance and reactance) of an alternating current of 20 or 100 kHz sent through the body. The method is based on the principle that different tissues have different resistances. Tissues with a high body water content, such as blood and muscles, have low resistance, while bone and fat tissue have high resistance. With BIA, body resistance is first measured, after which this measured resistance is converted into fat and muscle mass using specific formulas [16]. Our pilot study showed that, in comparison to the gold standard measure for body composition (air-displacement plethysmography (BODPOD)), which is time-consuming, expensive, and impossible to use in a home situation, the Inbody scale has good test–retest reliability but moderate validity [17]. The Inbody scale was provided for participants. Participants were asked to use the scales daily for two weeks, with the averages from these two weeks being used to obtain a more reliable estimate. This would be repeated at each measurement point. The data from the scales was automatically uploaded in the Inbody account of each participant connected to this scale. Data from the Inbody accounts were then merged with the PRO data from the questionnaires and clinical data from the NCR.

### Food intake

In large-scale epidemiological studies, food frequency questionnaires have been the preferred method for estimating the population’s long-term habitual food intake, mainly due to cost-effectiveness and logistical ease. Although the calculation of the food frequency questionnaires is automated and the results regarding nutritional intake are obtained quickly and easily, the disadvantage of the food frequency questionnaires is that it often overestimates nutritional intake and is susceptible to recall and social desirability bias [18, 19]. In smaller studies, a 3- or 7-day food diary is therefore preferred [20]. These food diaries are less susceptible to these biases but involve a lot of work for both the participant and the researcher because the food intake needs to be calculated manually afterwards. Other possibilities are web-based tools. Studies have shown that web-based tools are well received by patients and significantly simplify logistics and reduce costs compared to paper-and-pencil diaries or interview-based assessments, making them advantageous for large-scale assessments [21]. However, web-based dietary tools also have potential drawbacks: they require participant computer literacy and internet access, and some users may find digital food logging interfaces difficult or time-consuming, which can affect compliance and data accuracy. These challenges have been noted in recent evaluations of online dietary assessment methods [22]. Currently, there is no gold standard method for measuring dietary intake in nutritional epidemiology [9].

To assess food intake within PROFILES, we made a connection between our PROFILES registry and the online food diary (‘Eetmeter’) from the Dutch ‘Voedingscentrum’, which is a valid and reliable food diary [23]. An online food diary is less burdensome and less error-prone for both participants and researchers compared to a paper-and-pencil diary [24]. Patients are asked to register all the food and drinks they take during two weekdays and one weekend day using the online food diary [25, 26]. The Eetmeter is connected to the Dutch Nutrients Database so the quantity of macro- and micronutrients is calculated immediately. Alcohol intake is assessed in all PROFILES studies using the same standardized questionnaire items, regardless of whether patients use the Eetmeter.

## Legal Matters

After choosing suitable objective measurement instruments for your study, it is important to check whether legal action is necessary before the start of the study. If you place a large order of measuring instruments, a tender may be required by your institute or government. In addition, a contract between your institute and the manufacturer of the instrument might be necessary. For instance, when the data from participants in your study are not only saved by you but also by the company that provides the measurement instrument. In addition, the Medical Ethical Review board will not only assess whether your study meets the ethical standards, but also whether the proposed instruments are safe to use and not too burdensome for participants. Considering the number of Inbody scales we purchased, a tender was not necessary. Legal advice on these matters is recommended to ensure appropriate arrangements for each specific situation.

For the tape measures, legal action was not necessary since the outcomes of the measurements were written down onto the questionnaires. All PROFILES studies are performed in accordance with the Declaration of Helsinki. All studies are reviewed and approved by certified Medical Ethical Committees, in our case the METC Brabant, the Netherlands. Finally, all participants sign informed consent.

Participants in our study were referred to the Eetmeter via a link in our PROFILES questionnaire where they had to register and make an Eetmeter account. In the Eetmeter, participants explicitly consented to the sharing of their data from the start of the study until the study was completed or until the participant withdraws their consent. From the moment a participant withdraws their consent, their data will no longer be included in the study. The Eetmeter itself does not automatically transfer any data; instead, all data were downloaded by the research team for analysis. Because the data are first saved at the ‘Voedingscentrum’ (for two years) before it is retrieved by PROFILES, legal agreements were established to ensure proper data handling and compliance with regulations.

## Logistics

**Fig. 1:**
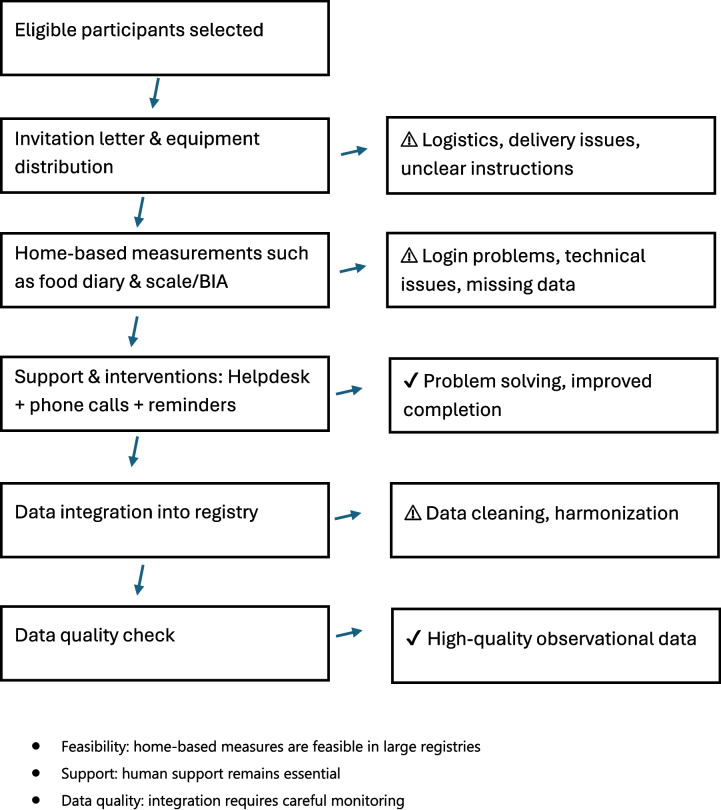
Schematic overview of the integration of home-based measurements into the PROFILES registry, illustrating the workflow from logistics to data integration and highlighting critical points where challenges occur and where supportive interventions improve feasibility and data quality

If the above-mentioned preconditions are ready, logistics are the next step to consider since participation in PRO studies is usually done completely remote (Fig. 1). Within our PROFILES studies, the Inbody scales were sent to participants' home address including a pre-stamped box so that patients could send the scale back to the PROFILES registry after use. In most cases, this went smoothly. However, some patients reported not having received a scale. Also, some participants did not return the scale to PROFILES, even after several reminders.

Response rates are also important to consider when discussing logistics. On the one hand, sending an instrument, like a weighing scale, to participants could increase response rates in a study if patients are interested in their own results. This especially is the case if they are allowed to keep the instrument after the study [27]. However, it could also decrease response rates since using a scale in combination with PRO questionnaires can be perceived as burdensome. Therefore, the use of scales was optional in our studies.

The tape measures were distributed to patients by including them in the information package that patients received about a study. Since not all potential participants decide to participate in a study, more tape measures were purchased beforehand compared to the number of participants we aimed to include.

While the logistics of online food diaries may appear straightforward, integrating them within the PROFILES registry required several technical adaptations. Specifically, an Application Programming Interface (API) had to be developed to enable secure data download from the Voedingscentrum to PROFILES. Participants were redirected to the Eetmeter registration page via a button embedded in the PROFILES environment, allowing them to create an Eetmeter account. The process required participants to first complete a PRO questionnaire, then follow a link to the online food diary, register for an account with the Voedingscentrum, confirm their account via email, and subsequently complete the diary over several consecutive days. Our pilot study showed that participants had difficulty creating an account. Once they had succeeded in doing so, filling in the food diary itself often posed no further problems. Placing the food diary at the very end of the questionnaire is not recommended, as some participants reported feeling less motivated to start with it at that point. A better approach is to provide the link to the Eetmeter separately. In one study, account creation was facilitated by helping participants directly at the hospital, which proved effective. Moreover, contacting patients by phone both before and after diary completion improved inclusion and adherence, although this was based on practical experience rather than on precise quantitative estimates.

## Instruction leaflets, videos, and helpdesk

Together with the home-use BIA scale, participants also received an instruction leaflet on how to use the scale. If they needed additional help with the scale, they could contact our PROFILES helpdesk (Fig. 1). Most of the phone calls on the scales were about solving issues with pairing the scale with a smart phone.

The instructions on the use of tape measures were included in both the online and paper-and-pencil questionnaires. No problems in using the tape measures were reported.

To enhance the number of participants using the “Eetmeter,” patients receive a phone call from a research assistant in which they were given instruction on how to create an ‘Eetmeter account’ and complete the food diary. We also made videos on our website that explain this. Despite this support, creating an ‘Eetmeter account’ that was paired with the patient’s PROFILES account was hard for a lot of participants, as confirmation of the account via email was required. Also, when the ‘Voedingscentrum’ updated their ‘Eetmeter,’ patients sometimes experienced problems with logging into the system. After completing their food intake, patients were called to check whether their data was complete. To support adherence, contacting participants by phone prior to completing their food diaries has proven to substantially improve participation even though this effect was not quantified with exact numerical data. In some of our studies, participants were only contacted after registering their food intake to verify whether they had completed the diary accurately. In other studies patients were not contacted.

## Collecting and merging the data

Data from home-use BIA scales were retrieved using the Inbody cloud-based system. For this study, we were provided with a dedicated Inbody account, which allowed us to access the measurements that participants had transmitted from their smartphones to the Inbody database. These data were subsequently exported to an Excel file for further processing. The linkage with the PROFILES registry was performed using the standard data integration procedures routinely applied within the registry framework.

The data on the tape measures was checked for outliers and then merged with the other data of the participants in the PROFILES registry.

Eetmeter data were automatically retrieved by PROFILES from the Voedingscentrum via a secure API and stored in the protected PROFILES environment. Access to this data was restricted to authorized researchers through secure credentials. The stored data included both the food items entered by participants and the calculated nutritional intake, summarized per meal and per day. These data were then merged with PRO data from questionnaires and clinical data from the NCR (Fig. 1).

## The value of adding objective data to PRO data

In all PROFILES studies, PRO data is combined with clinical data from the NCR. Since this clinical data is collected by trained registrars among everyone diagnosed with cancer in the Netherlands, these data are complete and reliable [28]. A shift was made from studies solely using questionnaires and clinical data to studies using a combination of questionnaires, clinical data, home-use BIA scales, online food diaries, and tape measures.

The combination of objective and subjective measures is crucial in studies on cancer survivors because it provides a holistic understanding of how cancer and its treatment impact survivors’ lives. Studies that combine both types of measures often provide a richer dataset, improving the ability to predict long-term outcomes and identifying risk factors for adverse physical or mental health outcomes. Also, adding objective data can improve accuracy and context. Subjective reports can sometimes misrepresent actual physical conditions (e.g., due to optimism bias or memory limitations). Objective data adds precision, helping to validate self-reported information and contextualize it in measurable terms. In addition, discrepancies between subjective and objective data can reveal important insights. Also, combining data sources can highlight underlying processes. For example, a participant may not have lost weight but still present with a low calf circumference, indicating a loss of muscle mass. The combination of both data types allows researchers and clinicians to design interventions and offer supportive care that address both the clinical needs (identified through objective measures) and personal needs (identified through subjective measures), leading to more personalized, effective care strategies.

With respect to our studies, being able to add objective data on body composition or food intake to the data we already have means that we can gain more detailed insight into physical health trajectories over time. For example, calf circumference measurements collected with measuring tapes allow us to monitor changes in muscle mass longitudinally, for instance in response to cancer treatment or nutritional interventions. Similarly, changes in fat mass can be observed—for example, a person may have a normal weight but an unfavorable fat distribution, which may be associated with poorer outcomes. Finally, longitudinal Eetmeter data enable us to track changes in energy and protein intake over time, making it possible to investigate whether lower protein intake is linked to worse clinical or psychosocial outcomes.

## The added value of normative data

While we do not yet have normative data for the home-use BIA scales or calf circumference measurements, reference percentiles for fat-free mass measured by BIA in healthy individuals are available [29], as well as established cut-off values for waist circumference [30]. In addition, a Dutch national food consumption survey provides updated reference data on dietary intake, which can be used to contextualize and interpret Eetmeter data [31]. Having access to such normative data is crucial for identifying deviations from expected values, distinguishing clinically relevant changes, and informing tailored interventions. It enables researchers and clinicians to evaluate whether a patient's body composition or dietary intake is within a normal range.

## The importance of sharing data

Since 2009, all PROFILES questionnaire data and aggregated clinical data have been available for noncommercial scientific research upon request to researchers worldwide (www.profilesregistry.nl). Soon, we will also make our objective data accessible. Sharing these resources not only enhances cancer survivor care, but by utilizing existing data, it reduces the burden on survivors who would otherwise need to participate in additional studies, making research funding more efficient. Additionally, we offer access to the PROFILES-registry application including objective measures, enabling other researchers to collect their data in a streamlined, efficient way.

## Conclusion

This paper highlights the challenges, considerations and critical insights gained from using home-use BIA scales, online food diaries and tape measures in a longitudinal population-based PROFILES registry study. Practical insights—such as the importance of selecting valid and user-friendly tools, ensuring proper logistics and data protection, and supporting participant engagement—can inform future research that aims to enrich PROs with objective and ambulatory measures.

Combining PRO data with objective indicators of body composition and nutritional intake offers a more comprehensive understanding of cancer survivorship. This approach enhances our ability to predict which survivors are at risk for poor outcomes, understand the underlying mechanisms, and monitor the effects of interventions over time. Such knowledge is essential not only for developing targeted, evidence-based interventions but also for improving clinical decision-making and personalized survivorship care. Ultimately, this contributes to the prevention and management of long-term and late effects of cancer and its treatment and supports survivors in maintaining or regaining optimal health and quality of life.

## Data Availability

PROFILES data is freely available upon request (www.profilesregistry.nl) for scientific purposes.

## Abbreviations

BIA: Bioelectric impedance analysis
BMI: Body mass index
HRQoL: Health-related quality of life
NCR: Netherlands Cancer Registry
PROFILES: Patient reported outcomes following initial treatment and long-term evaluation of survivorship
PROs: Patient-reported outcomes
QoL: Quality of life
